# scTIME: single-cell-informed interpretable modeling of immunotherapy-associated immune states in non-small cell lung cancer

**DOI:** 10.3389/frai.2026.1918104

**Published:** 2026-10-06

**Authors:** Guozhi Chen, Chenqiu You, Hong Gao, Chengjie Xu

**Affiliations:** 1 YongJia People's Hospital, Wenzhou, China; 2 Shanxi Acupuncture Hospital, Taiyuan, China; 3 Department of General Surgery, Sir Run Run Shaw Hospital, Zhejiang University School of Medicine, Hangzhou, China

**Keywords:** immunotherapy, interpretable machine learning, multi-omics, non-small cell lung cancer, single-cell RNA sequencing, tumor immune microenvironment

## Abstract

Immune checkpoint inhibitor response in non-small cell lung cancer (NSCLC) reflects coordinated tumor and immune states that are not fully captured by single biomarkers. We developed scTIME, an interpretable model that translates single-cell immune programs into bulk transcriptomic scores, and integrated public single-cell, T-cell receptor, transcriptomic, survival, and proteogenomic cohorts. Response modeling used two cohorts with different endpoints (40 patients) and compared six algorithms. ElasticNet was selected as a sparse reporting model, achieving a pooled five-fold out-of-fold ROC-AUC of 0.810; nested model selection yielded 0.772. Matched held-out stratification identified response-enriched scTIME groups in a direct descriptive comparison with CD274, while the fitted score emphasized cytotoxic CD8, natural killer, SPP1 macrophage, interferon-response, and antigen-presentation programs. Single-cell analyses and external projections linked these programs to CXCL10-CXCR3 recruitment, exclusion-associated states, and T-cell receptor ecology across molecular contexts. Exploratory survival and cross-omics analyses extended the framework beyond the response cohorts but were interpreted as projection rather than independent validation. Together, the results show how cell-resolved immune biology can support transparent, multi-omic biomarker development without reducing response to a single pathway. scTIME remains exploratory because model choice was *post hoc*, cohorts were small and clinically heterogeneous, and endpoint-matched external validation was unavailable.

## 1 Introduction

Non-small cell lung cancer (NSCLC) is one of the solid tumors in which immune checkpoint inhibitors (ICIs) have been most widely implemented, and PD-1/PD-L1 blockade has reshaped the management of advanced and resectable disease. First-line immunotherapy improves outcomes in selected patients with advanced NSCLC, although benefit varies by regimen and clinical subgroup (Ferrara et al., 2021). Neoadjuvant nivolumab plus chemotherapy has also improved event-free survival and pathological complete response in resectable disease (Forde et al., 2022). PD-L1 remains a common stratification marker but does not fully explain treatment response (Uruga and Mino-Kenudson, 2021). Contemporary biomarker reviews emphasize that tumor mutational burden, immune infiltration, inflammatory transcriptional programs, and microenvironmental features should be considered together (Wang X. et al., 2023). Resistance studies likewise indicate that checkpoint blockade response is not determined by a single pathway (Pavelescu et al., 2024). Integrated genomic and transcriptomic profiling has linked response to both tumor-intrinsic states and the immune microenvironment (Ravi et al., 2023). The central precision-immunotherapy challenge is therefore to define composite tumor-immune states rather than another isolated positive biomarker.

Public patient-derived molecular datasets provide heterogeneous clinical and multi-omics observations that can complement conventional trial evidence in exploratory biomarker research. Such research nevertheless requires explicit provenance, endpoint definitions, and analytical boundaries (Luo and Solit, 2025). Recent guidance emphasizes transparent reporting of regression and machine-learning prediction models (Collins et al., 2024). Multimodal biomedical AI is most informative when molecular layers are treated as complementary views (Acosta et al., 2022). Accordingly, we do not label molecular projections as independent validation sets. *Post hoc* model explanations do not by themselves establish reliability or mechanism (Ghassemi et al., 2021). We therefore treat GEO, TCGA, and CPTAC as public patient-derived research resources, not as estimates of real-world clinical effectiveness.

The tumor immune microenvironment provides a biologically coherent framework for understanding ICI response in NSCLC. Antigen presentation, interferon signaling, T-cell priming, myeloid regulation, and stromal remodeling jointly shape treatment sensitivity (Cascone et al., 2022). The efficacy of checkpoint blockade depends on dynamic interactions among tumor, immune, and stromal compartments (Genova et al., 2021). NSCLC can evade T-cell-mediated killing through multiple layered mechanisms (Xiang et al., 2024). Acquired resistance is also accompanied by microenvironmental remodeling and functional restriction of T cells (Wang et al., 2025). Strategies for converting cold tumors into hot tumors consequently need to consider both T-cell recruitment and exclusion pressure (Yu et al., 2025).

Single-cell RNA sequencing and spatial omics provide high-resolution foundations for dissecting the NSCLC immune ecosystem. Single-cell analysis after neoadjuvant immunotherapy has revealed treatment-associated remodeling that is difficult to resolve with bulk RNA sequencing alone (Hu et al., 2023). A large atlas of anti-PD-1-treated NSCLC demonstrated substantial interpatient immune heterogeneity linked to pathological response (Liu et al., 2025). Integrated single-cell and spatial analyses further indicate that treatment-associated changes involve both cell states and tissue organization (Cui et al., 2025). Neoantigen-specific tumor-infiltrating lymphocytes show that functional T-cell state can be more informative than total T-cell abundance (Caushi et al., 2021). Cell-state resolution can also refine NSCLC classification and patient stratification (Leader et al., 2021). Contemporary multimodal single-cell methods provide a general framework for integrating complementary cellular measurements (Hao et al., 2021).

NSCLC immune ecology is additionally shaped by histology, lineage composition, and spatial organization. Lung adenocarcinoma and squamous cell carcinoma can carry distinct single-cell immune backgrounds (Wang et al., 2022). Intratumoral plasma cells illustrate that non-T-cell compartments may also carry treatment-outcome information (Patil et al., 2022). Lung tumor-infiltrating regulatory T cells comprise divergent states linked to checkpoint blockade response (Dykema et al., 2023). Cancer-associated fibroblast phenotypes are associated with NSCLC outcome (Cords et al., 2024). Their spatial positioning and matrix programs can promote T-cell exclusion in human lung tumors (Grout et al., 2022). Macrophage localization and state may likewise influence sensitivity or resistance to anti-PD-1/PD-L1 therapy (Larroquette et al., 2022). Spatial multi-omics signatures reinforce the need to integrate tissue structure with molecular immune programs (Aung et al., 2025). Pan-cancer single-cell analyses further show that tumor-normal ecosystem comparisons require cell-state-aware interpretation (Kang et al., 2024).

Artificial intelligence and machine learning are increasingly used in health research, but their value depends on clinical context, transportability, and transparent evaluation rather than technical complexity alone (Rajpurkar et al., 2022). Deep-learning models have demonstrated potential for immunotherapy-response stratification in advanced NSCLC (Rakaee et al., 2025). CT-based ensemble learning suggests that noninvasive imaging can carry information related to ICI benefit (Saad et al., 2023). Multimodal CT models have also been trained against pathological response after neoadjuvant immunochemotherapy (Ye et al., 2024). Machine-learning assessment of tumor-infiltrating lymphocytes can convert routine histology into response-associated features (Rakaee et al., 2023). Weakly supervised whole-slide learning provides a further pathology-based approach to major pathological response prediction (Han et al., 2024). The high prevalence of bias in supervised machine-learning prediction studies makes fold-local preprocessing and independent validation especially important (Andaur Navarro et al., 2021).

Based on this rationale, we developed single-cell-informed Tumor Immune MicroEnvironment modeling (scTIME), an exploratory framework for extracting immunotherapy-response insights from public multi-omics resources. Tumor-intrinsic IFN-alpha and CXCL10 can promote T-lymphocyte recruitment and activation (Cheng et al., 2024). IFN-gamma-mediated induction of CXCL10 and ICAM-1 can enhance activation of CXCR3-high CD8 T cells in lung cancer (Wang C. L. et al., 2023). Increased intratumoral CXCL10 has also been linked experimentally to anti-PD-L1 activity (Huang et al., 2023). Engineered CXCL9 and CXCL10 variants further support the translational relevance of CXCR3-ligand biology (Lugassy et al., 2025). Tumor-intrinsic innate signaling can reshape the microenvironment for checkpoint therapy (Vornholz et al., 2023). RGS1-mediated regulation of the ATF3-IFNGR1 axis provides another example of tumor-intrinsic control of checkpoint response (Wang B. et al., 2023). These studies motivated contextual analyses; they were not treated as proof that CXCL10-CXCR3 dominates the fitted classifier.

## 2 Materials and methods

### 2.1 Public cohorts and analytical roles

This exploratory public-data study integrated single-cell RNA-seq, TCR, bulk RNA-seq, TCGA, and CPTAC resources to construct scTIME, a single-cell-informed model of NSCLC immune states. The design followed the principle of translating cell-type-specific programs into interpretable bulk features (Turan et al., 2023). Response modeling used GSE126044 radiologic anti-PD-1 response (*n* = 16) and GSE207422 bulk neoadjuvant major pathological response (*n* = 24), harmonized only as a pragmatic benefit/MPR label. GSE207422 single-cell comparisons retained 4 MPR/pCR and 10 non-MPR samples after excluding one not-evaluable sample (Hu et al., 2023). GSE243013 provided single-cell immune-state and TCR ecology analyses (Liu et al., 2025). GSE135222 was used for exploratory PFS projection, and the deposited GSE274975 expression metadata were restricted to histology-level description because patient-level ICI outcomes were unavailable (Poddubskaya et al., 2024). TCGA-LUAD/LUSC and CPTAC LUAD/LSCC were used only for external molecular projection; CPTAC LSCC has been characterized proteogenomically (Satpathy et al., 2021).

### 2.2 Single-cell state and TCR analysis

GSE207422 embeddings, labels, and markers mapped major tumor, immune, myeloid, fibroblast, endothelial, and mast-cell states. Annotations were checked against marker programs and recent lung single-cell ecosystem studies (Ren et al., 2024). SPP1-positive tumor-associated macrophages were retained as a myeloid feature rather than assumed to be uniformly suppressive (Almgrami et al., 2025). GSE243013 composition analyses retained 233 anti-PD-1-treated patients with evaluable pathology, including 125 MPR/pCR and 108 non-MPR cases. TCR analyses retained 221 patients with clonality summaries, including 121 MPR/pCR and 100 non-MPR cases. Patient-level cell fractions were compared by two-sided Mann–Whitney U tests with Benjamini-Hochberg correction, and clonality-composition relationships used Spearman correlation.

### 2.3 Immune module scoring and bulk projection

Twelve modules were scored from single-cell marker programs and immunotherapy-related biology: IFN response, antigen presentation, cytotoxic CD8, progenitor exhausted CD8, terminal exhausted CD8, Treg, SPP1 macrophage, TGF-beta/EMT, hypoxia, NK, CXCL10-CXCR3 axis, and CD274 expression. Module scores were projected into bulk RNA-seq, TCGA, and CPTAC matrices when genes were available. Values were standardized within cohorts before PCA, response-association testing, and model fitting. In every held-out analysis, scaling parameters were estimated only from training rows and then applied to held-out rows.

### 2.4 scTIME model development and evaluation

Six candidate models were benchmarked on the 40 labeled samples: ElasticNet logistic regression, ridge logistic regression, random forest, radial-basis-function support-vector machine, XGBoost, and LightGBM. ElasticNet was retained as the reporting model because its mixed L1/L2 penalty gives a sparse signed logit, but this *post hoc* choice was not treated as selection-independent. Reporting followed TRIPOD+AI recommendations for transparent prediction-model studies (Collins et al., 2024). PROBAST+AI motivated explicit separation of development, selection, and applicability claims (Moons et al., 2025). Pooled ElasticNet evaluation used one five-fold out-of-fold prediction per patient with fold-local preprocessing. A fixed-seed nested sensitivity analysis used five stratified outer folds and four inner folds for candidate selection. Reciprocal analyses trained on one response cohort and tested on the other, with training-cohort scaling only, and were interpreted according to external-validation guidance (Riley et al., 2024). Performance summaries included ROC-AUC, PR-AUC, Brier score, calibration, bootstrap intervals, and decision-curve analysis. Decision curves were treated as exploratory threshold-specific net benefit (Vickers et al., 2023). The final refit on all 40 samples was used only for coefficients, patient-level linear contributions, and external projections.

### 2.5 Contextual immune biology

CXCL10-CXCR3, IFN response, antigen presentation, cytotoxic CD8, and CD274 modules were compared between benefit/MPR and no-benefit/non-MPR samples by two-sided Mann–Whitney U tests with Benjamini-Hochberg correction. The IFN-antigen index averaged IFN-response and antigen-presentation scores and was related to CXCL10-CXCR3 by Spearman correlation. Single-cell CXCL9/CXCL10/CXCL11-CXCR3 interaction and ligand-activity scores were treated as inferred communication rather than measured binding (Jin et al., 2021). TGF-beta/EMT, hypoxia, fibroblast-associated features, Treg, and terminally exhausted CD8 were evaluated as contextual exclusion biology, not as a fitted counter-axis. This interpretation followed evidence on TGF-beta-associated immune suppression (Matsuda et al., 2023). Hypoxia was retained as external context (Robles-Oteiza et al., 2025). TGF-beta-activated fibroblast context was retained separately (Hou, 2025). The SPP1 signature used SPP1, APOE, TREM2, LIPA, CD68, and MARCO, with SPP1-CD44, SPP1-integrin, and ligand-activity screens. Bulk composition was estimated with a local nonnegative least-squares reference workflow and interpreted cautiously because deconvolution varies by method (Merotto et al., 2025).

### 2.6 Held-out stratification and external projection

CD274 was analyzed as a standalone benchmark because its final ElasticNet coefficient was zero. scTIME stratification used pooled five-fold out-of-fold probabilities, and scTIME and CD274 were split within each cohort by matched top-versus-bottom quartiles and upper-versus-lower median halves before pooling counts. Response enrichment was assessed with two-sided Fisher exact tests, without a formal paired superiority claim. GSE135222 PFS projection used a continuous-score Cox model, a median-split Kaplan–Meier analysis with a log-rank test, and an age/sex-adjusted sensitivity model. TCGA analyses used primary-tumor patient-level scores and excluded invalid survival times. CPTAC proteome and phosphoproteome layers were analyzed separately for cross-omics and stage-associated projections. GSE274975 remained descriptive histology-only projection. All external analyses were treated as molecular context rather than ICI-response validation.

## 3 Results

### 3.1 Public multi-omics framework

We first organized the public patient-derived resources around a simple analytical sequence: define immune states at single-cell resolution, project those states into bulk transcriptomic cohorts, evaluate an interpretable response score, and then use external omics resources only for contextual projection. Figure 1 summarizes this workflow, while Table 1 separates response-modeling cohorts from survival, histology, TCGA, CPTAC, single-cell, and TCR resources.

**Figure 1:**
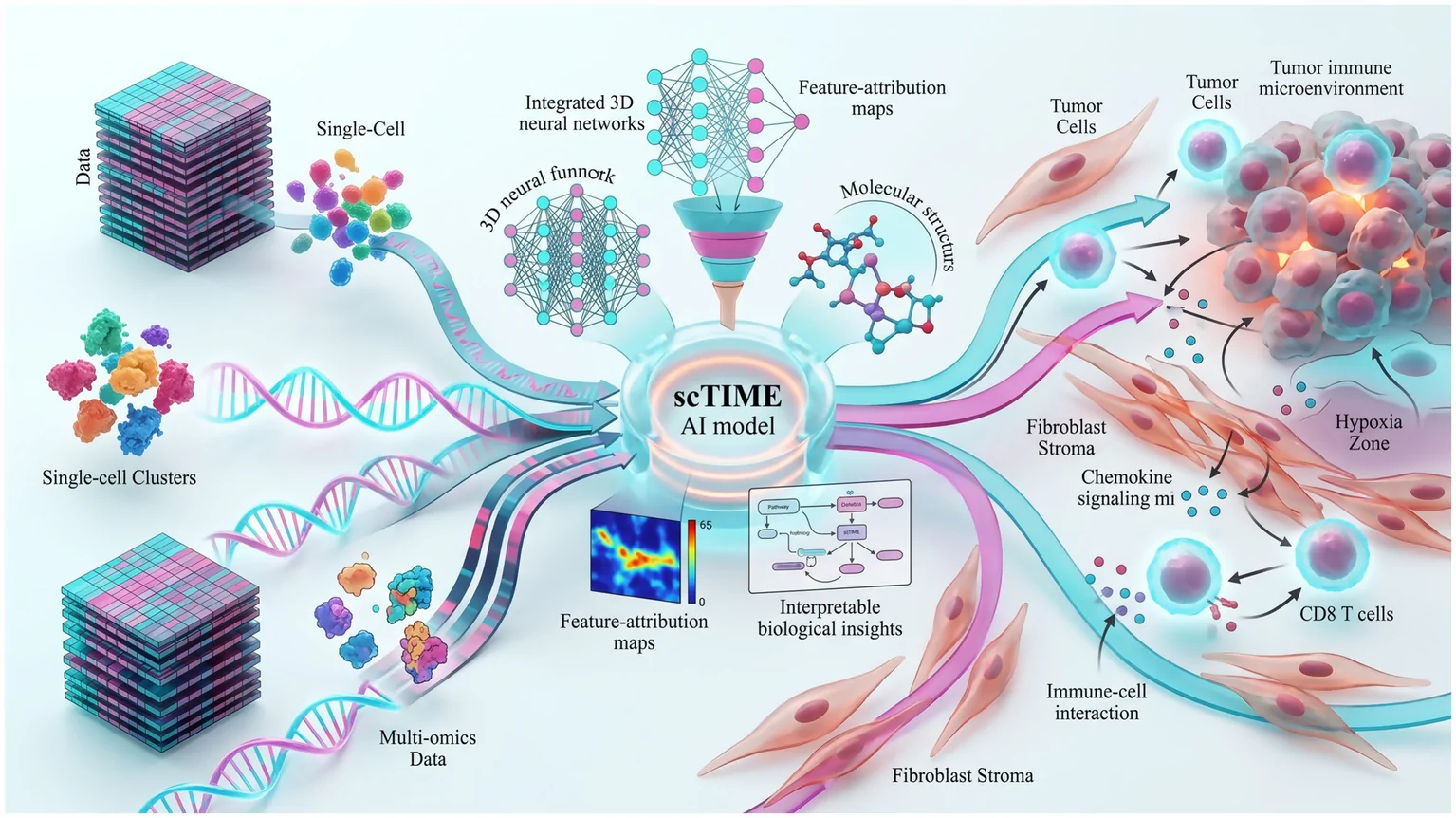
Study framework of scTIME modeling and evaluation.

**Table 1:** Cohort resource summary.

Cohort	Modality	Samples	Role
GSE126044	Bulk RNA-seq	16	Response modeling; radiologic anti-PD-1 response
GSE207422 bulk	Bulk RNA-seq	24	Response modeling; pathologic response after neoadjuvant therapy
GSE207422 single-cell	scRNA-seq	15 samples; 14 evaluable (4 MPR/pCR, 10 NMPR)	Single-cell ligand-receptor context; NE excluded from response tests
GSE135222	Bulk RNA-seq	27	Exploratory PFS projection only
GSE274975	Bulk RNA-seq	61	Histology-only projection; no ICI outcome
GSE243013 single-cell	scRNA-seq	243 total; 233 analyzed (125 MPR/pCR, 108 non-MPR)	Cell-state response analysis; no-anti-PD-1 and unknown-response records excluded
GSE243013 TCR	TCR-seq	231 total; 221 response-evaluable	TCR immune-ecology analysis
TCGA-LUAD	Bulk RNA-seq	516 primary-tumor patients; 503 valid OS records	Primary-tumor patient-level non-ICI projection and exploratory OS association
TCGA-LUSC	Bulk RNA-seq	501 primary-tumor patients; 493 valid OS records	Primary-tumor patient-level non-ICI projection and exploratory OS association
CPTAC-LSCC	Proteome and phosphoproteome	108 specimens; 216 omics-layer records	Non-ICI cross-omics projection; layers analyzed separately
CPTAC-LUAD	Proteome and phosphoproteome	110 specimens; 220 omics-layer records	Non-ICI cross-omics projection; layers analyzed separately

This separation is central to the interpretation of the study. GSE126044 and GSE207422_bulk were the only cohorts used for response modeling, whereas GSE135222, GSE274975, TCGA, CPTAC, and GSE243013 served distinct biological or projection roles. The source-data manifest in Supplementary Table S1 links each plotted panel to its machine-readable input, allowing the main text to focus on biological findings rather than file-level provenance.

### 3.2 Single-cell immune ecology

The single-cell analyses showed that NSCLC immunotherapy response was organized across multiple immune compartments rather than by one isolated cell type. The GSE207422 map resolved major tumor, immune, stromal, and vascular states, and marker-program checks supported the annotations used for downstream module construction (Figure 2A; Supplementary Figures S2A,B).

**Figure 2:**
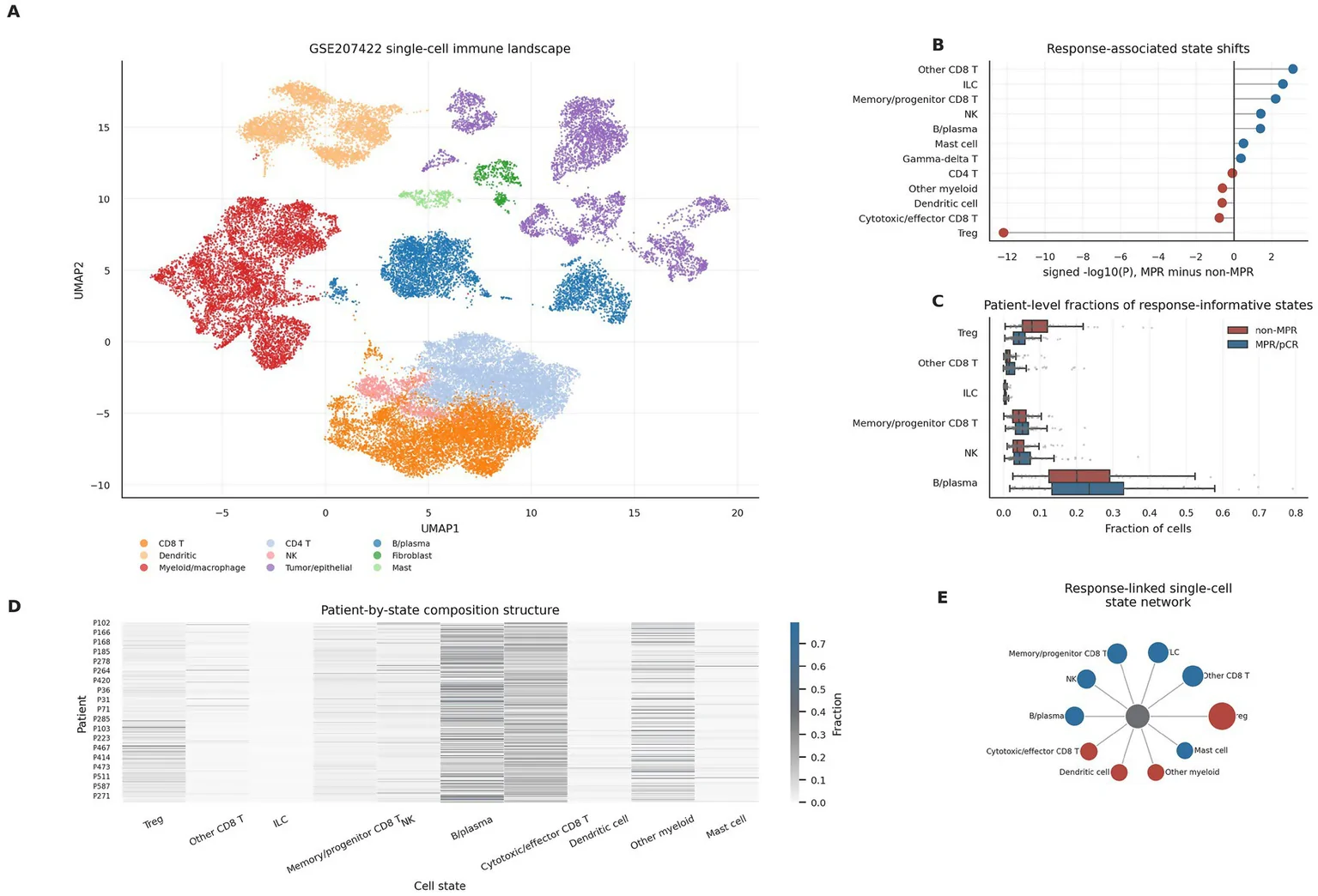
Single-cell response landscape in NSCLC immunotherapy cohorts. **(A)** GSE207422 single-cell UMAP. **(B)** GSE243013 state fractions. **(C)** Patient-level response-informative state fractions. **(D)** Patient-by-state composition heatmap. **(E)** Response-linked single-cell state network.

In GSE243013, response-associated composition shifts involved effector-oriented lymphoid states as well as suppressive context. MPR/pCR samples showed enrichment of CD8-related and innate lymphoid states, whereas Treg enrichment was more characteristic of non-MPR samples (Figure 2B; Supplementary Table S2). Patient-level fraction plots and the response-linked state network then showed that these differences were distributed heterogeneously across individuals, supporting a composite immune-ecology interpretation rather than a single-marker narrative (Figures 2C–E).

### 3.3 Exploratory scTIME modeling

We next asked whether the single-cell-informed modules could be translated into a bulk RNA-seq score. Projection into immune-module space placed labeled and external samples in a common descriptive coordinate system (Figure 3A). Among the six candidate algorithms, ElasticNet and ridge had the same rounded pooled out-of-fold ROC-AUC, so ElasticNet was retained for its sparse signed coefficient vector rather than presented as a uniquely superior model (Figure 3B; Table 2).

**Figure 3:**
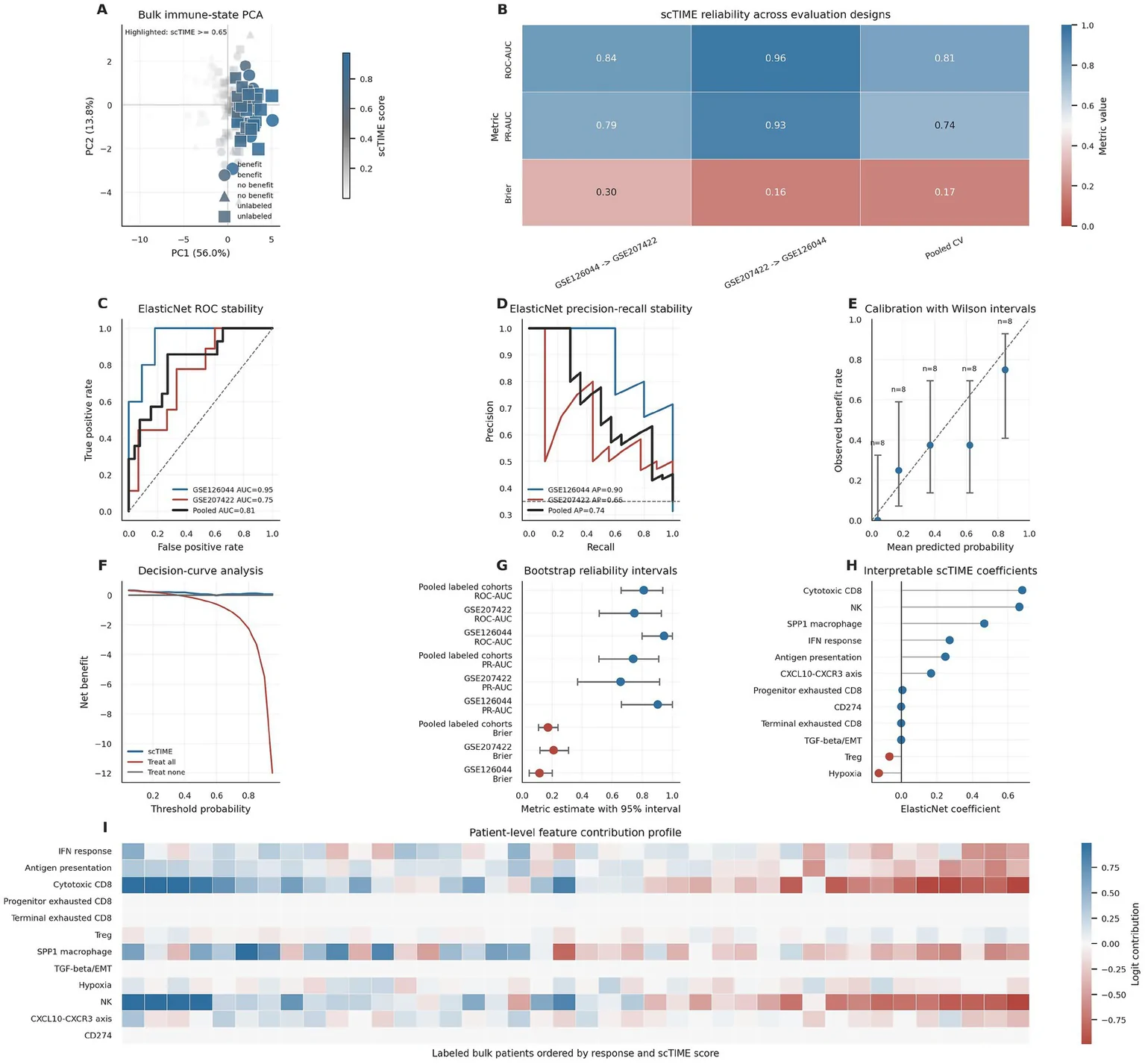
Reliability, evaluation and interpretability of scTIME. **(A)** PCA projection of bulk samples in immune-module space. **(B)** ElasticNet ROC-AUC, PR-AUC, and Brier score across pooled out-of-fold and reciprocal-cohort evaluations. **(C)** out-of-fold ROC curves. **(D)** out-of-fold precision-recall curves. **(E)** Calibration curve. **(F)** decision-curve analysis. **(G)** Bootstrap reliability intervals. **(H)** All 12 final ElasticNet coefficients, including zero coefficients. **(I)** full-refit patient-level feature contributions for interpretation.

**Table 2:** Main model validation metrics.

Analysis	Model_or_score	Evaluation_context	n	Benefit_mpr	Roc_auc	Pr_auc	Brier	Group_or_cutoff	Benefit_mpr_count	Benefit_mpr_rate	Two_sided_fisher_p	Score_provenance	Interpretation
Candidate benchmark	ElasticNet logistic	Pooled five-fold CV with dataset-specific scaling estimated in each training fold	40	14	0.81044	0.739899	0.17299					One prediction per patient from a fold model and fold-fitted preprocessing	Retained sparse interpretable model
candidate benchmark	Ridge logistic	Pooled five-fold CV with dataset-specific scaling estimated in each training fold	40	14	0.81044	0.749135	0.183437					One prediction per patient from a fold model and fold-fitted preprocessing	Candidate benchmark only
Candidate benchmark	Random forest	Pooled five-fold CV with dataset-specific scaling estimated in each training fold	40	14	0.785714	0.622264	0.179526					One prediction per patient from a fold model and fold-fitted preprocessing	Candidate benchmark only
candidate benchmark	RBF SVM	Pooled five-fold CV with dataset-specific scaling estimated in each training fold	40	14	0.725275	0.497515	0.200396					One prediction per patient from a fold model and fold-fitted preprocessing	Candidate benchmark only
Candidate benchmark	XGBoost	Pooled five-fold CV with dataset-specific scaling estimated in each training fold	40	14	0.711538	0.571108	0.22404					One prediction per patient from a fold model and fold-fitted preprocessing	Candidate benchmark only
Candidate benchmark	LightGBM	Pooled five-fold CV with dataset-specific scaling estimated in each training fold	40	14	0.721154	0.590595	0.315113					One prediction per patient from a fold model and fold-fitted preprocessing	Candidate benchmark only
nested selection sensitivity	Inner-selected candidate	Five outer folds; four inner folds; preprocessing fit inside every training fold	40	14	0.771978	0.628536	0.189396					Nested outer held-out	Selected model counts: RandomForest = 3; Ridge_logistic = 1; XGBoost = 1
Reciprocal cohort sensitivity	ElasticNet logistic	GSE207422_bulk training to GSE126044 test; preprocessing fit in training cohort	16	5	0.963636	0.926667	0.162438					Held-out cohort	Different response endpoint and treatment context from the training cohort
Reciprocal cohort sensitivity	ElasticNet logistic	GSE126044 training to GSE207422_bulk test; preprocessing fit in training cohort	24	9	0.837037	0.791733	0.300098					Held-out cohort	Different response endpoint and treatment context from the training cohort
pooled-CV subgroup sensitivity	ElasticNet logistic	GSE126044 subset of pooled five-fold OOF predictions	16	5	0.945455	0.902857	0.117093					Pooled five-fold out-of-fold	Subgroup sensitivity; not leave-one-cohort-out validation
Pooled-CV subgroup sensitivity	ElasticNet logistic	GSE207422_bulk subset of pooled five-fold OOF predictions	24	9	0.748148	0.655668	0.210255					Pooled five-fold out-of-fold	Subgroup sensitivity; not leave-one-cohort-out validation
Held-out stratification	scTIME	Within-cohort quartile groups then pooled	10	7				Top_quartile	7	0.7	0.003096	Pooled_5fold_out_of_fold_ElasticNet	High_or_top; identical split scheme used for scTIME and CD274
Held-out stratification	scTIME	Within-cohort quartile groups then pooled	10	0				Bottom_quartile	0	0	0.003096	Pooled_5fold_out_of_fold_ElasticNet	Low_or_bottom; identical split scheme used for scTIME and CD274
held-out stratification	CD274	Within-cohort quartile groups then pooled	10	5				Top_quartile	5	0.5	0.140867	Within_cohort_expression	High_or_top; identical split scheme used for scTIME and CD274
held-out stratification	CD274	Within-cohort quartile groups then pooled	10	1				Bottom_quartile	1	0.1	0.140867	Within_cohort_expression	Low_or_bottom; identical split scheme used for scTIME and CD274
Held-out stratification	scTIME	Within-cohort median groups then pooled	20	12				High_median_half	12	0.6	0.0022	Pooled_5fold_out_of_fold_ElasticNet	High_or_top; identical split scheme used for scTIME and CD274
held-out stratification	scTIME	Within-cohort median groups then pooled	20	2				Low_median_half	2	0.1	0.0022	Pooled_5fold_out_of_fold_ElasticNet	low_or_bottom; identical split scheme used for scTIME and CD274
Held-out stratification	CD274	Within-cohort median groups then pooled	20	8				High_median_half	8	0.4	0.741054	Within_cohort_expression	High_or_top; identical split scheme used for scTIME and CD274
Held-out stratification	CD274	Within-cohort median groups then pooled	20	6				Low_median_half	6	0.3	0.741054	Within_cohort_expression	Low_or_bottom; identical split scheme used for scTIME and CD274

The pooled ElasticNet ROC-AUC was 0.810, while the fixed nested model-selection sensitivity yielded ROC-AUC 0.772. These values support an exploratory signal but also show why the model should not be treated as selection-independent. Reciprocal cohort-held-out analyses, calibration, decision-curve analysis, and bootstrap intervals are therefore presented as reliability context rather than as clinical validation (Figures 3C–G; Table 2).

The fitted score was biologically interpretable. Figure 3H reports all 12 final coefficients: cytotoxic CD8 and NK modules dominated the positive logit, SPP1 macrophage, IFN response, antigen presentation, and CXCL10-CXCR3 contributed positively, hypoxia and Treg were the only negative coefficients, and terminally exhausted CD8, TGF-beta/EMT, and CD274 were exactly zero. Patient-level contribution plots were used only to interpret the final refit and not as held-out explanations (Figure 3I; Supplementary Table S3).

### 3.4 Activation and CXCL10-CXCR3 programs

The positive coefficient pattern pointed to a hot immune-state signal rather than a single dominant chemokine mechanism. CXCL10-CXCR3 was higher in benefit/MPR samples as a response-associated trend, whereas antigen presentation and cytotoxic CD8 showed stronger positive shifts (Figure 4A). Across the 40 labeled samples, the IFN-antigen index was strongly linked to CXCL10-CXCR3 activity; Spearman correlation reached rho = 0.736 with *p* = 6.31e-08, supporting coupling between IFN response, antigen presentation, and CXCR3-ligand biology (Figure 4B).

**Figure 4:**
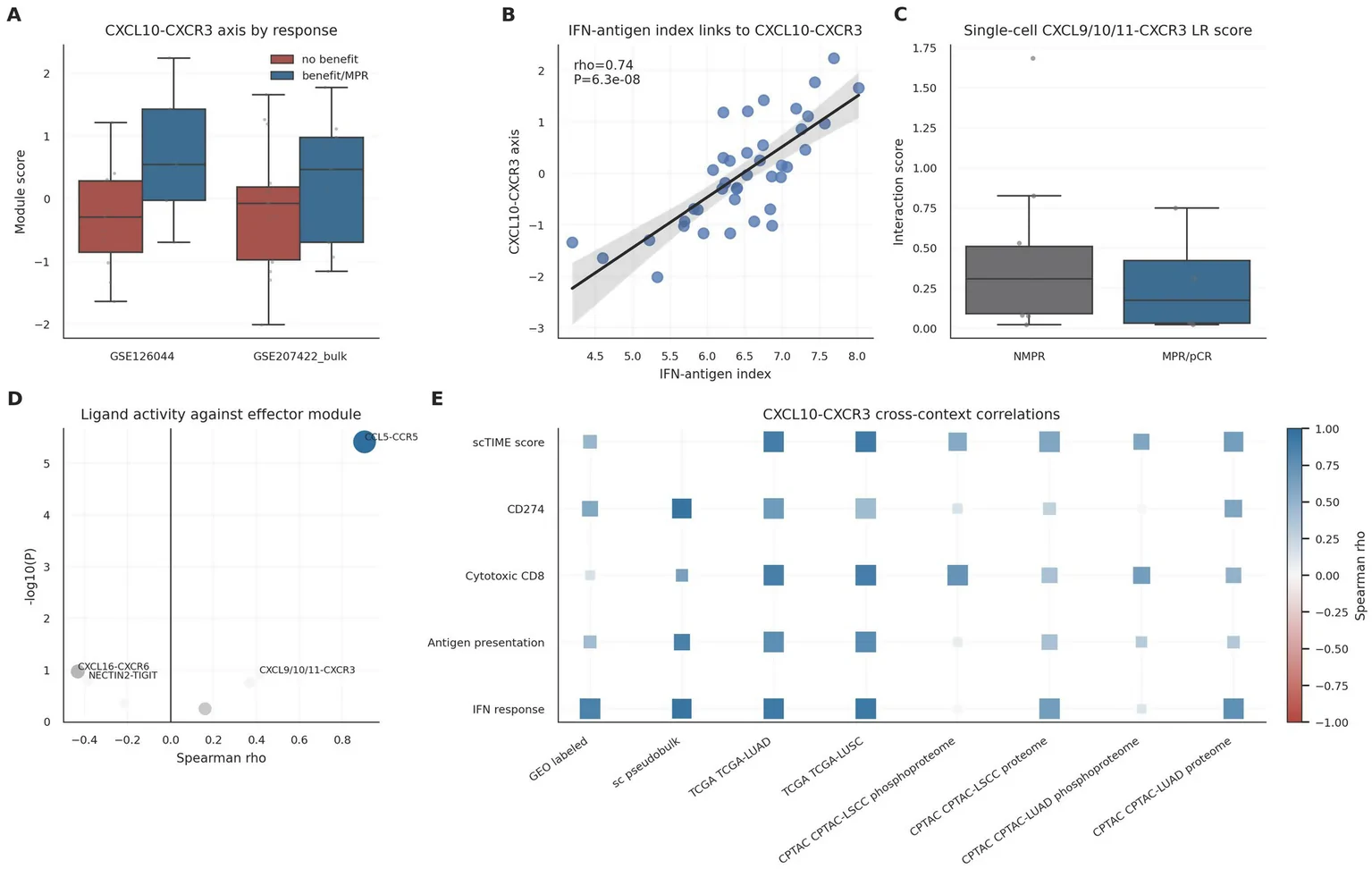
CXCL10-CXCR3 and IFN-antigen axis. **(A)** CXCL10-CXCR3 axis comparison. **(B)** IFN-antigen index correlation. **(C)** Single-cell CXCL9/CXCL10/CXCL11-CXCR3 interaction score. **(D)** Ligand activity against the effector module. **(E)** Cross-context axis correlations.

Single-cell ligand-receptor and ligand-activity analyses placed this axis in a broader communication landscape rather than elevating it to a causal mechanism. The CXCL9/CXCL10/CXCL11-CXCR3 score localized the prespecified recruitment axis, while the ligand-activity screen highlighted additional effector-associated interactions (Figures 4C,D; Supplementary Figure S2C).

Cross-context analyses reinforced the same interpretation. In labeled GEO samples, CXCL10-CXCR3 correlated with IFN response (rho = 0.853), antigen presentation (rho = 0.428), cytotoxic CD8 (rho = 0.168), and CD274 expression (rho = 0.581) (Figure 4E). These relationships support an activation-recruitment program, but they do not establish prospective biomarker performance or causal recruitment.

### 3.5 Contextual exclusion biology

We then evaluated TGF-beta/EMT, hypoxia, fibroblast-associated features, Treg, terminally exhausted CD8, and SPP1 macrophage as context for interpreting the immune-state score. This analysis was deliberately separate from coefficient interpretation: TGF-beta/EMT and CD274 were zero-coefficient features, hypoxia and Treg were small negative components, and fibroblast fraction was not part of the fitted model (Figure 5A).

**Figure 5:**
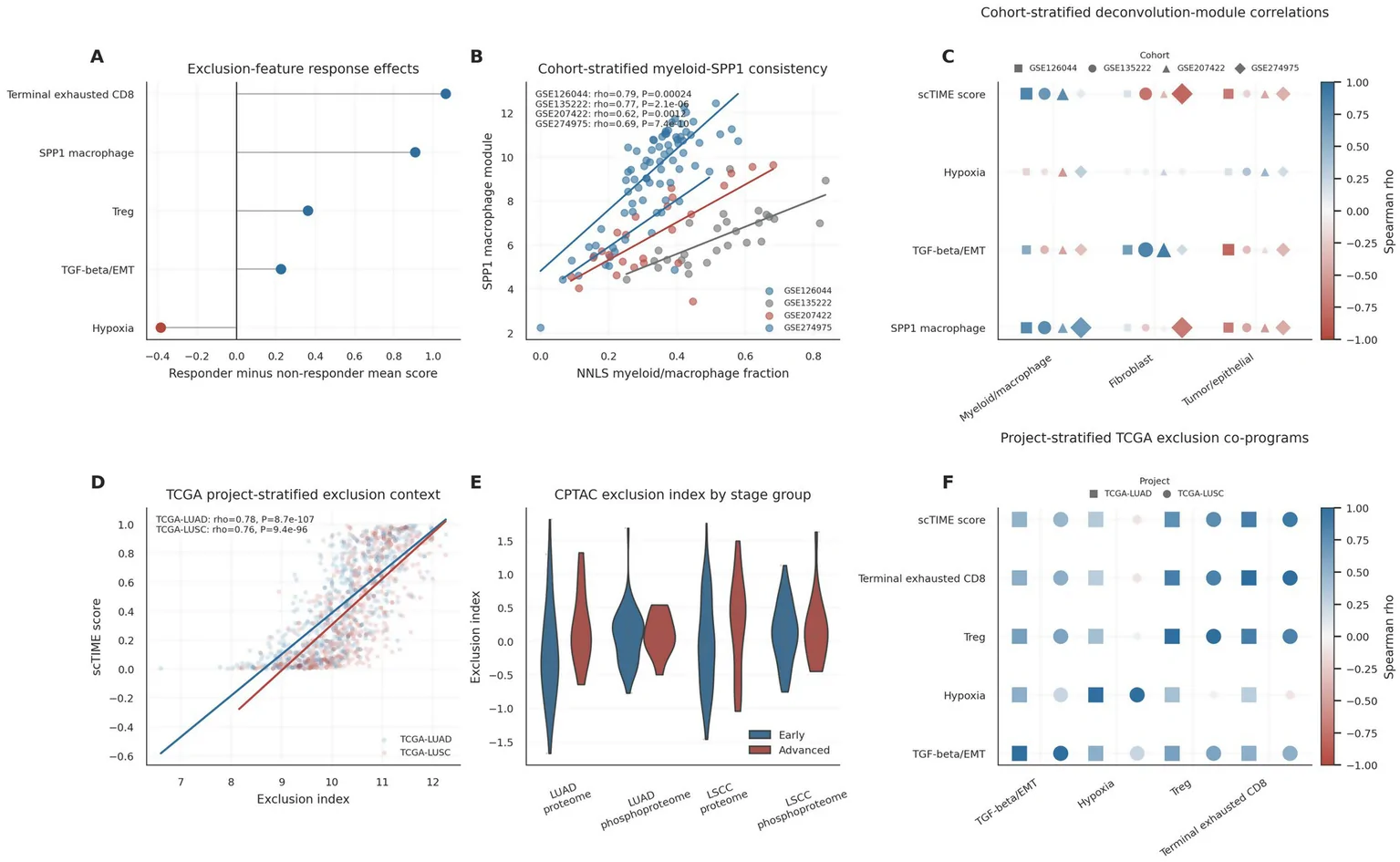
TGF-beta/EMT, hypoxia and immune-context programs. **(A)** Separately analyzed contextual and myeloid features. **(B)** cohort-stratified relationships between NNLS myeloid/macrophage fraction and the SPP1 macrophage module. **(C)** cohort-stratified deconvolution-cell fraction correlations. **(D)** project-stratified relationships between the TCGA contextual-exclusion composite and scTIME. **(E)** CPTAC contextual-exclusion composite. **(F)** TCGA within-project co-program structure. The composite excludes SPP1 and fibroblast fraction; TGF-beta/EMT is a zero-coefficient feature in scTIME, and these panels do not establish a fitted counter-axis.

SPP1 illustrated why this separation matters. The bulk SPP1 macrophage module aligned with responder-like immune ecology and with myeloid/macrophage deconvolution within cohorts, whereas single-cell SPP1 interaction and ligand-activity analyses trended toward a non-MPR context without surviving FDR correction (Figure 5B; Supplementary Figures S2D,E). The result is best read as an abundance-versus-interaction hypothesis, not as evidence for a causal switch.

The remaining contextual analyses were consistent with that cautious framing. Fibroblast and exclusion-associated programs showed cohort- and project-dependent relationships, so pooled cross-project effects were not inferred (Figures 5C–F). TCGA and CPTAC projections describe molecular co-programs in non-ICI resources; they do not test treatment response and do not convert contextual exclusion biology into a fitted counter-axis.

### 3.6 Held-out stratification and TCR ecology

Because PD-L1 remains a common clinical reference point, we compared CD274 with scTIME using identical within-cohort cutoffs. scTIME stratification used one out-of-fold probability per labeled patient, whereas CD274 was analyzed as a standalone marker because its ElasticNet coefficient was zero (Figure 6A).

**Figure 6:**
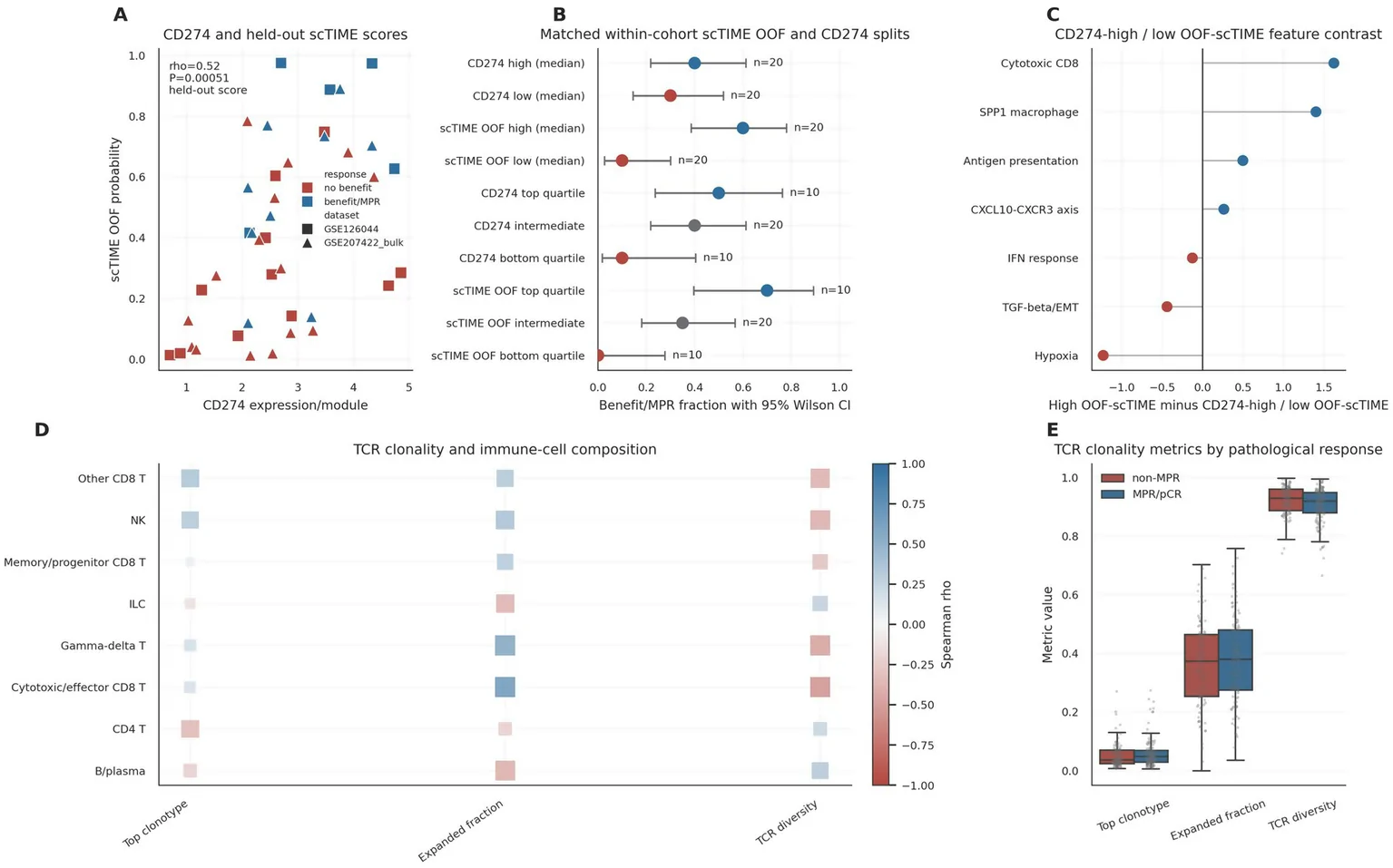
Held-out scTIME and CD274 stratification with TCR immune ecology. **(A)** CD274 and one pooled five-fold out-of-fold scTIME probability per labeled sample. **(B)** Response counts after applying matched cutoffs within each cohort and then pooling groups. For top versus bottom quartiles, scTIME contained 7/10 versus 0/10 benefit/MPR cases (two-sided Fisher *p* = 0.00310), whereas CD274 contained 5/10 versus 1/10 (*p* = 0.141). For upper versus lower median halves, scTIME contained 12/20 versus 2/20 benefit/MPR cases (*p* = 0.00220), whereas CD274 contained 8/20 versus 6/20 (*p* = 0.741). **(C)** Exploratory feature contrasts based on held-out score strata. **(D)** TCR clonality and immune-cell composition. **(E)** TCR clonality in 221 patients with an evaluable response and clonality summary (121 MPR/pCR and 100 non-MPR). The response eligibility filter excluded nine records without documented anti-PD-1 treatment and one with unknown pathological response; 12 additional response-evaluable composition patients lacked TCR clonality summaries. CD274 is shown as a standalone comparator because its fitted scTIME coefficient is zero; no formal paired test of biomarker superiority was performed.

With matched quartile and median cutoffs, scTIME-defined high groups were more response-enriched than the corresponding CD274 groups. Exact response counts and Fisher tests are reported in Figure 6B, its legend, and Table 2 so that the Results can emphasize interpretation: this is a small-sample descriptive comparison, not a formal paired superiority test.

Feature contrasts across held-out score strata supported the same hot immune-state pattern without implying that scTIME adds information through CD274 itself (Figure 6C). In GSE243013, expanded clonotypes tracked with effector CD8, gamma-delta T, and NK fractions, but response-associated TCR clonality metrics did not survive FDR correction (Figures 6D,E). TCR ecology therefore remained supportive biological context rather than an input to scTIME.

### 3.7 External survival and multi-omics projection

Finally, we projected the same module definitions beyond the response-modeling endpoints. In GSE135222, scTIME showed an exploratory association with improved PFS, and the age/sex-adjusted sensitivity analysis gave a similar direction. Because smoking history, performance status, stage, and treatment-line information were unavailable, this result was interpreted as projection rather than independent prognostic or response validation (Figures 7A,B).

**Figure 7:**
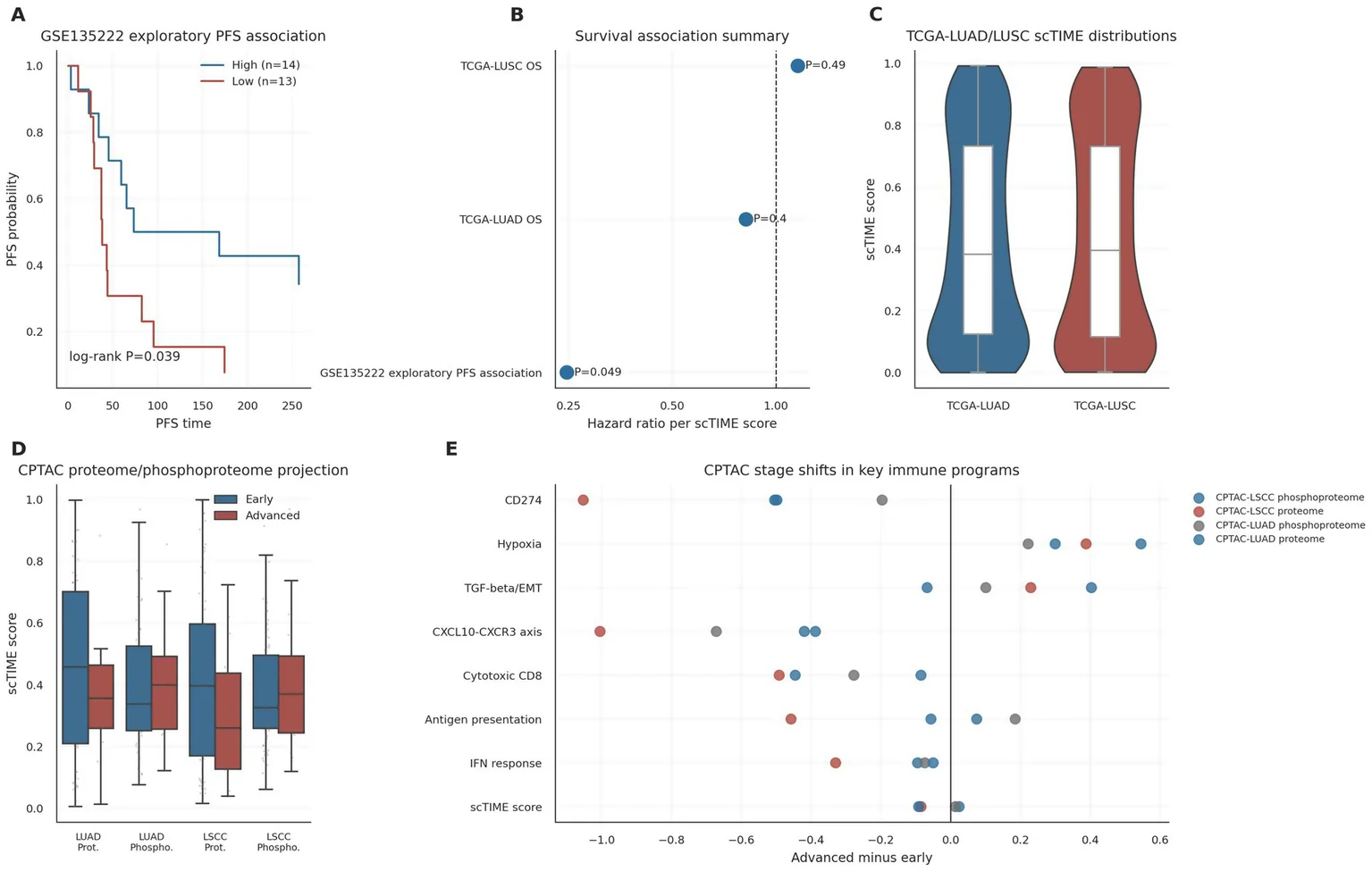
Exploratory PFS association and cross-omics projection of scTIME. **(A)** Exploratory Kaplan–Meier PFS association in GSE135222. **(B)** Survival association summary. **(C)** TCGA-LUAD/LUSC score distributions. **(D)** CPTAC score projection by stage. **(E)** CPTAC stage-associated immune-program shifts. GSE274975 is reported in the text and source data as descriptive histology-only projection, not response validation.

TCGA-LUAD/LUSC primary-tumor projections showed broad score variation but no significant overall-survival association, reinforcing their role as non-ICI molecular context (Figures 7B,C). CPTAC proteome and phosphoproteome projections were likewise used to describe cross-omics and stage-associated immune programs, not treatment response (Figures 7D,E). The deposited GSE274975 expression metadata used here did not provide patient-level ICI-response outcomes, so its histology-level scTIME projection was descriptive only. Together, these external analyses show computational portability of the scoring framework, not clinical transportability of the probability estimates.

## 4 Discussion

This study frames scTIME as a single-cell-informed and interpretable model for exploratory immunotherapy-response insight generation in NSCLC. Its contribution is the integration of single-cell discovery, bulk response modeling, held-out evaluation, cross-omics projection, and explicit coefficient auditing. Transparent reporting is particularly important when model choice follows comparison of several candidates on the same small dataset (Collins et al., 2024). Prediction-model risk-of-bias guidance also requires model selection to be distinguished from independent validation (Moons et al., 2025). ElasticNet was retained because its mixed penalty provides a compact signed score in the presence of correlated modules, not because it was a stable universal winner. The nested analysis selected random forest in three folds, ridge in one, and XGBoost in one, so model-selection uncertainty accompanies the reported sparse coefficient vector. Interpretability alone cannot convert an internally evaluated association into a clinical mechanism (Ghassemi et al., 2021).

The findings are consistent with the broader view that NSCLC response is a composite immune state rather than a single marker. Neoadjuvant single-cell data demonstrate treatment-associated remodeling at both cell-state and patient levels (Hu et al., 2023). Larger single-cell atlases likewise show substantial heterogeneity after anti-PD-1-based therapy (Liu et al., 2025). In our held-out descriptive analysis, scTIME quartile and median splits showed stronger response enrichment than matched CD274 splits, but the sample size was only 40 and no formal paired superiority test was performed. CD274 was among the candidate features but its coefficient was exactly zero, so the comparison is with a clinically familiar standalone marker rather than evidence of incremental information within a CD274-containing logit. Reciprocal cohort tests provide transportability context, but they do not substitute for a sufficiently powered external validation study (Riley et al., 2024).

The fitted score was dominated by cytotoxic CD8 and NK modules, with SPP1 macrophage, IFN response, and antigen presentation also positive. CXCL10-CXCR3 was a smaller positive component, while hypoxia and Treg were the only negative coefficients. TGF-beta/EMT, terminally exhausted CD8, and CD274 were exactly zero. Acquired-resistance studies support treating exclusion and immune activation as evolving context rather than a fixed binary mechanism (Memon et al., 2024). Hypoxia can impair checkpoint sensitivity and alter interferon-induced chemokine biology (Yabuki et al., 2025). Pharmacologic perturbation can also change MHC-I and PD-L1 expression, cautioning against interpretation of CD274 in isolation (Tu et al., 2022). SPP1 macrophage abundance was higher in benefit/MPR samples and carried a positive coefficient, whereas SPP1 ligand-receptor scores trended in the opposite direction without surviving false-discovery correction. This discordance supports an abundance-versus-interaction hypothesis but does not identify a causal switch or coefficient sign change.

Several limitations remain. GSE126044 measured radiologic response to anti-PD-1 therapy, whereas GSE207422 measured major pathological response after neoadjuvant treatment, so the pooled label is a pragmatic harmonization rather than a common endpoint. The model used 12 candidate modules but included only 14 positive labels among 40 patients, leaving coefficients, thresholds, and discrimination vulnerable to instability. The six-model screen was non-nested, the nested estimate was lower, and supervised machine-learning research shows that reuse of development data can produce optimistic performance (Andaur Navarro et al., 2021). The GSE135222 PFS association remains susceptible to unmeasured smoking, performance-status, stage, and treatment-regimen confounding despite age and sex adjustment. Bulk projection cannot reconstruct spatial cell contact, and cancer-state deconvolution remains dependent on reference and modeling assumptions (Murillo et al., 2023). Deep-learning deconvolution offers a complementary strategy but does not remove the need for matched biological validation (Xiong et al., 2024). TCGA and CPTAC lack ICI outcomes, and the SPP1 abundance-versus-interaction contrast remains hypothesis-generating. Independent endpoint-matched cohorts, preregistered thresholds, spatial validation, and complete computational provenance are required before clinical or mechanistic claims are made.

## 5 Conclusion

This study establishes scTIME as a single-cell-informed and interpretable framework for exploratory immune-state modeling in NSCLC immunotherapy. In held-out analyses, it separated response-enriched groups in two small, endpoint-different cohorts and was dominated by cytotoxic CD8/NK and other hot-tumor modules. CXCL10-CXCR3 and IFN-antigen programs were associated contextual features, whereas TGF-beta/EMT, terminal exhaustion and CD274 contributed zero to the fitted logit. TGF-beta/EMT, hypoxia and fibroblast programs should therefore be treated as external biological hypotheses, not as validated model counterweights. Prospective endpoint-matched cohorts are required before scTIME can support treatment selection or mechanistic claims.

## Data Availability

The datasets presented in this study can be found in online repositories. The names of the repository/repositories and accession number(s) can be found in the article/Supplementary material.

## Glossary

AI: Artificial intelligence
AUC: Area under the curve
CAF: Cancer-associated fibroblast
CPTAC: Clinical Proteomic Tumor Analysis Consortium
EMT: Epithelial-mesenchymal transition
FDR: False discovery rate
ICI: Immune checkpoint inhibitor
IFN: Interferon
LUAD: Lung adenocarcinoma
LSCC: Lung squamous cell carcinoma
MPR: Major pathological response
NSCLC: Non-small cell lung cancer
PFS: Progression-free survival
PR-AUC: Precision-recall area under the curve
ROC-AUC: Receiver operating characteristic area under the curve
TAM: Tumor-associated macrophage
TCGA: The Cancer Genome Atlas
TCR: T-cell receptor
TGF-beta: Transforming growth factor-beta
TME: Tumor microenvironment
